# The effect of lysolecithin on prostanoid and platelet-activating factor formation by human gall-bladder mucosal cells

**DOI:** 10.1155/S0962935195000147

**Published:** 1995-03

**Authors:** M. K. Nag, Y. G. Deshpande, D. Beck, A. Li, D. L. Kaminski

**Affiliations:** 1 Department of Surgery St Louis University Health Sciences Center 3635 Vista Ave. at Grand Blvd P.O. Box 15250 St Louis MO 63110-0250 USA

## Abstract

It has been demonstrated that lysolecithin (lysophosphatidyl choline, LPC) produces experimental cholecystitis in cats mediated by arachidonic acid metabolites. LPC is a cytolytic agent that has been postulated as a contributing factor in the development of cholecystitis in humans. The purpose of this research was to evaluate the effect of LPC on human gall-bladder mucosal cell phospholipase A_2_ and cyclooxygenase activity. Gall-bladder mucosal cells were isolated from the gall-bladders of patients undergoing routine cholecystectomy. Fresh, isolated cells were maintained in tissue culture and stimulated with varying doses of LPC. Platelet-activating factor concentration was quantitated as an index of phospholipase A_2_ activity and prostanoids were measured as an index of cyclooxygenase activity. Also, the effect of LPC on cyclooxygenase 1 and 2 expression in microsomal protein was evaluated. LPC caused dose related increases in 6-keto-PGF_1α_ and PAF produced by human gall-bladder mucosal cells. Exposure of human gall-bladder mucosal cells to LPC failed to elicit expression of constitutive cyclooxygenase-1, while the expression of inducible cyclooxygenase-2 was increased. The results of this study indicate that LPC induces the formation of prostanoids and PAF by human gall-bladder mucosal cells, suggesting that this substance may promote the development of gall-bladder inflammation.


Mediators of Inflammation 4, 90–94 (1995)

### The effect of lysolecithin on prostanoid and platelet-activating factor formation by human gall-bladder mucosal cells

M. K. Nag, Y. G. Deshpande, D. Beck, A. Li and D. L. Kaminski[CA]

Department of Surgery, St Louis University Health Sciences Center, 3635 Vista Ave. at Grand Blvd, P.O. Box 15250, St Louis, MO 63110-0250, USA

[CA] Corresponding Author

**It has been demonstrated that lysolecithin (lysophosphatidyl choline, LPC) produces experimental cholecystitis in cats mediated by arachidonic acid metabolites. LPC is a cytolytic agent that has been postulated as a contributing factor in the development of cholecystitis in humans. The purpose of this research was to evaluate the effect of LPC on human gall-bladder mucosal cell phospholipase $A_2$ and cyclooxygenase activity. Gall-bladder mucosal cells were isolated from the gall-bladders of patients undergoing routine cholecystectomy. Fresh, isolated cells were maintained in tissue culture and stimulated with varying doses of LPC. Platelet-activating factor concentration was quantitated as an index of phospholipase $A_2$ activity and prostanoids were measured as an index of cyclooxygenase activity. Also, the effect of LPC on cyclooxygenase 1 and 2 expression in microsomal protein was evaluated. LPC caused dose related increases in 6-keto-$PGF_{1\alpha}$ and PAF produced by human gall-bladder mucosal cells. Exposure of human gall-bladder mucosal cells to LPC failed to elicit expression of constitutive cyclooxygenase-1, while the expression of inducible cyclooxygenase-2 was increased. The results of this study indicate that LPC induces the formation of prostanoids and PAF by human gall-bladder mucosal cells, suggesting that this substance may promote the development of gall-bladder inflammation.**

**Key words**: Cyclooxygenase, Gall-bladder, Human cholecystitis, Lysolecithin, Mucosal cell, Platelet-activating factor, Prostanoids

### Introduction

Lysolecithin (lysophosphatidyl choline, LPC) is present in human bile in patients with cholecystitis and has long been implicated in the pathogenesis of cholecystitis.[1–6] In animals LPC instilled into the lumen of the gall-bladder produces gall-bladder inflammation with the process mediated by eicosanoids.[7] Platelet-activating factor (1-alkyl-2-acetyl-*SN*-glycero-3-phosphocholine, PAF) is a purported mediator of inflammation[8] and has been found to produce experimental cholecystitis in animals.[9]

The purpose of this study was to evaluate the effect of LPC on human gall-bladder mucosal cell prostanoid and PAF formation. Prostanoids and PAF are both products of the phospholipase $A_2$ metabolic pathway.[10] It was anticipated that demonstrating alterations in PAF formation would suggest that LPC produces its effect by stimulating phospholipase $A_2$ activity, as well as by altering cyclooxygenase activity.

### Materials and Methods

With the approval of the St Louis University Institutional Review Board as well as informed consent, 17 patients undergoing routine cholecystectomy took part in this study. The mean (± S.E.M.) age of the seven males and ten females was 47 (± 3) years. All specimens were opened in the operating room and the mucosal surface evaluated by a pathologist to ensure that the gall-bladder had no areas that were indicative of neoplasms. Stones were present in seven of the gall-bladders, while ten did not have stones and were removed incidentally at the time of liver or pancreatic resections. Six of the gall-bladders with stones were removed by laparoscopy. The gall-bladders used were selected to obtain specimens with minimal inflammation and thus provide greater mucosal cell yields.

Gall-bladder mucosal cell isolation and culture were performed as described previously.[11] Gall-bladder specimens were maintained in 0.15 M NaCl solution on ice and underwent isolation of mucosal cells within 30 min of operative removal. Blood and bile were washed from the specimen with cold Hanks' balanced salt solution (HBSS). The gall-bladder was incubated with HBSS without calcium and magnesium (Sigma, St Louis, MO, USA) in a 0.25% trypsin (Sigma), 0.25% EDTA (Sigma) solution for 45 min at 37°C, while shaking in a water bath (Precision Scientific, Princeton, NJ, USA; speed 5). The mucosal surface was gently scraped with a scalpel blade. The cells were dispersed with pipette suction and disper-

© 1995 Rapid Communications of Oxford Ltd sion. The suspension was centrifuged twice at 100 × **g** for 5 min at 4°C. The cell pellet was mixed with 50 ml minimum essential medium (Sigma, St Louis) with 10% foetal bovine serum (SMEM) and centrifuged at 1000 × **g** for 10 min. A 50 ml Percoll solution was prepared with SMEM according to the density formula and the cell pellet was suspended in the solution and centrifuged at 1400 × **g** for 10 min to remove red blood cells. Subsequently, 25 ml of the supernatant was removed and mixed with 25 ml of SMEM and centrifuged at 2000 **g** for 10 min. The pellet was washed again with 25 ml medium and centrifuged. The cells were suspended in 10 ml of medium and counted. Using Trypan Blue exclusion, viability was invariably greater than 90%. The cells were inoculated onto 35 mm type I collagen coated culture dishes (Co-star, Cambridge, MA, USA) at a seeding density of 1 × $10^6$ cells/well.

Cells were cultured in 5% $CO_2$ 95% air environment at 37°C in minimum essential medium containing 10% heat inactivated serum with 292 mg/l L-glutamine, 100 mg/l streptomycin sulphate. 100 000 IU/l penicillin G and 2.5 mg/l amphotericin B (all from Sigma). As evaluated by phase contrast microscopy, the attached cell population was uniform without evidence of blood cells. Immunoperoxidase techniques[11,12] were used to characterize gall-bladder epithelial cell specific antigens with avidin–biotin-complex conjugated antisera (Vecta Stain ABC kit, Vector Laboratories, Burlingame, CA, USA). Vimentin monoclonal antibody was used as the negative control. Employing cytokeratin 19 (monoclonal anti-cytokeratin 19, Amersham Corp., Arlington Heights, IL, USA), immunohistochemical staining approximately 90% of the cell population stained positively.

Gall-bladder mucosal cells were evaluated after 24 h in culture to abrogate differences in the inflammatory characteristics of the gall-bladder present at the time of cholecystectomy. In addition, each gall-bladder served as its own control. Depending on the mucosal cell yield, 12, 24 or 36 wells were utilized/gall-bladder. Attached cells were washed twice with 10 ml Krebs–Ringer buffer (KRB, Sigma, St Louis), and incubated for 1 h in 1 ml KRB solution in 95% $O_2$ 5% air atmosphere. When appropriate LPC (Sigma) was added to the incubation medium in concentrations of 0.1, 0.25, 0.5, 1.0 and 1.5 mM and the cells incubated for 1 h at 37°C. These LPC concentrations are within the range of LPC concentrations present in human bile and in experimental animal models of gallstone formation.[2,3,6,13]

Following completion of the incubation period the cells and incubation buffer were separated and the buffer centrifuged to remove any cells and then subsequently frozen at –70°C prior to assay. The attached cells were washed with fresh KRB and 1 ml collagenase solution (Sigma) was added to each well and incubated for 20 min. The cells were scraped from the wells, centrifuged at 200 × **g** for 10 min and the pellet was stored frozen at –70°C. Cell protein was determined by the method of Bradford[14] on mucosal cell specimens which were solubilized with 0.1 N NaOH for 1 h at 37°C and sonicated for 10 s. Bovine albumin was used as the standard.

The buffer was thawed and aliquots were used for PGE, 6-keto-$PGF_{1\alpha}$ and PAF radioimmunoassays performed in buffer samples without extraction, using commercially available RIA kits obtained from New England Nuclear, Dupont, Boston, MA.[11,15] Without extraction, the RIA employed measured PGE, 6-keto-$PGF_{1\alpha}$ and PAF added to buffer solution in a linear manner with increasing concentrations between 50 pg and 5 ng. The PGE antibody does not discriminate well between $PGE_1$ and $PGE_2$. The 6-keto-$PGF_{1\alpha}$ antibody exhibited 2.6% cross-reactivity with $PGF_{2\alpha}$. The PAF assay employed cross-reacts equally with $C_{16}$ and $C_{18}$ PAF and acyl-PAF, but not with lyso-PAF.[11,15]

Lactate dehydrogenase (LDH) concentrations in buffer samples were determined in order to estimate the effects of LPC on gall-bladder mucosal cell integrity. Using cell free supernatants, LDH activity was measured by spectrophotometry using NADH and sodium pyruvate as substrates.[16]

Using three separate gall-bladder specimens, attempts were made to determine if LPC stimulated cyclooxygenase-1 or -2 (COX-1 or COX-2) enzyme expression. Following 24 h of incubation attached cells were washed twice with 10 ml KRB buffer and incubated for 1 h in 1 ml KRB solution in 95% $O_2$, 5% air atmosphere at 37°C with and without LPC (0.5 mM). After 1 h, the buffer was discarded and the cells were washed with PBS, detached and collected. After centrifugation at 2500 **g** the pellets were resuspended in 5 ml 0.1 M Tris-HCl pH 8.0 containing 10 mM diethylthiocarbamic acid, 5 mM EDTA, 250 μM leupeptin, 0.1 μg/ml chymostatin, 2 μg/ml aprotinin, 1 μg/ml pepstatin A, 7.2 μg/ml E-64, 2.5 μg/ml antipapain, and 0.1 ng/ml benzamidine (all from Sigma). Cells were sonicated three times for 20 s each and then centrifuged again for 20 min at 10 000 × **g** at 4°C. The supernatant was then centrifuged again for 2 h at 100 000 × **g** at 4°C. The microsomal pellet was resuspended in 0.5 ml of 0.1 M phosphate buffer pH 8.0 and the protein concentration was determined.

For Western blotting 15 μg of gall-bladder microsomal protein per specimen was separated by SDS–polyacrylamide gel electrophoresis.[17] All the samples were boiled for 5 min in SDS–PAGE sample buffer containing 2% SDS, 20% v/v β-mercaptoethanol and 0.1% bromophenol blue, before loading onto the gel wells. The electrophoresis was carried out using the Bio-Rad electrophoresis apparatus (Bio-Rad, Rockeville Center, NY, USA). The gel was run at 100 V for 2 h.

The polypeptides were then transferred using electrophoresis onto nitrocellulose membranes, using the Bio-Rad transblot apparatus.[18] Transfer was performed at 30 V for 16 h in 25 ml Tris-HCl pH 8.3, 192 mM glycine and 20% methanol.

The nitrocellulose membranes were treated employing the Promega Biotec technique (Promega Biotec, Madison, WI). The membranes were transferred into a solution containing 10 ml of TBS-T (containing 100 mM Tris–HCl, pH 8.0, 150 mM NaCl and 0.5% Tween-20) and rinsed briefly to remove any remnants of acrylamide. The rinsed membranes were blocked by incubating with 10 ml of TBS-T containing 1% BSA at room temperature for 30 min with gentle shaking. The membranes were then incubated separately with polyclonal anti-COX-1-goat antibody (Cayman, Ann Arbor, MI, diluted 1:100) and polyclonal anti-COX-2-rabbit antibody (Oxford Biomedical, Oxford, MI, diluted 1:100) at room temperature for 3 h with gentle shaking. As positive controls, 100 ng of COX-1 (Cayman) or COX-2 protein (Oxford) was added to the membranes. The nitrocellulose membranes were then washed with TBS-T three times for 10 min each to remove unbound antibodies.

Subsequently, the membranes were transferred into solutions containing 7.5 ml of secondary antibodies and incubated at room temperature for 1 h. For COX-1 detection rabbit anti-goat IgG alkaline phosphatase conjugate (Bio-Rad, 1:3000 dilution) was employed and for COX-2 goat anti-rabbit IgG alkaline phosphatase conjugate (Bio-Rad, 1:3000 dilution) was used as the secondary antibody. The membranes were then washed with 200 ml of TBS-T three times as before. The membranes were blotted on damp filter paper and transferred to 5 ml of colour development substrate solution consisting of 33 μl of NBT substrate containing 50 mg/ml in a 70% solution of *N,N*-dimethylformamide (Sigma) added to 5 ml of alkaline phosphatase buffer containing 100 mM Tris-HCl pH 9.5, 100 mM NaCl, 5 mM $MgCl_2$ and 16.5 μl of 5-bromo-4 chloro-3-indolyl phosphate (BCIP, Sigma). After the colour had developed to the desired intensity, the reaction was stopped by replacing the substrate solution with stop solution (20 mM Tris–HCl pH 8.0 and 5 mM EDTA).

All samples were evaluated in duplicate. Statistical analysis was performed using analysis of variance. When the $F$ value was significant, differences between mean values were evaluated employing the least significant difference. As used throughout this study, significance indicates $p < 0.05$.

### Results

The effect of varying concentrations of LPC on gall-bladder mucosal cell integrity was evaluated in a variety of ways. LDH is a lysosomal enzyme commonly measured in tissue culture media as an index of the amount of cellular contents lost through damaged cell membranes.[16] As seen in Table 1, there was a statistically significant increase in the concentrations of LDH in cells exposed to LPC compared with cells maintained only in control buffer solution. Whereas the LDH changes suggested that exposure to LPC may produce some cell damage, when cell morphology, attachment to basement membrane substrate or Trypan Blue exclusion were evaluated, we were unable to detect any differences between control cells in buffer solution and cells exposed to 1.5 mM concentrations of LPC.

As seen in Table 1, LPC produced dose-related increases in 6-keto-$PGF_{1\alpha}$ and PAF concentrations in buffer solution, while PGE concentrations were unchanged. Results are presented by reference to $1 \times 10^6$ cells and were similar when evaluated by reference to mg cell protein. The results suggested that LPC was a significant stimulant of the hydrolysis of the *SN*-2 fatty acid chain of phospholipids leading to the formation of PAF and of COX activity producing arachidonic acid metabolites.

To obtain further evidence that LPC stimulated synthesis of COX, microsomal proteins were fractionated by SDS polyacrylamide gel electrophoresis, electroblotted on nitrocellulose filters and treated with anti-COX-1 and anti-COX-2 antibodies. In control buffer solutions and in buffer solutions from LPC stimulated gall-bladder mucosal cells COX-1 protein was not detectable or barely detectable suggesting that the expression of the constitutive COX-1 enzyme was not significantly increased (Fig. 1). As seen in Fig. 2, LPC treatment induced increased expression of inducible COX-2 as demonstrated by the rabbit anti-COX-2 antibody. These results support the conclusion that LPC stimulates *de novo* synthesis of 72 kDa inducible COX-2 enzyme[19,20] in human gall-bladder mucosal cells.

### Discussion

The results of the present study suggest that a cytolytic, membrane perturbing substance present in bile,[4] LPC, produces significant changes in COX and phospholipase $A_2$ metabolism by human gall-bladder mucosal cells. While the changes in LDH levels indicate some limited membrane damage may be occurring,[21] the evidence suggests that LPC has direct stimulatory activity on phospholipase $A_2$ and COX enzymes. Support for this conclusion is related to the specificity of the changes in PAF and prostanoid formation with increased prostacyclin formation and the increased expression of inducible COX-2 in response to LPC stimulation.

COX-1 protein was not detectable or barely detectable and did not appear to be inducible by LPC. This is true in other systems as well. In skin inflammation,

**Table 1.** The effect of lysophosphatidylcholine (LPC) on prostanoid and PAF production and LDH release by human gall-bladder mucosal cells.

| | LPC (mM) | | | | | |
|---|---|---|---|---|---|---|
| | Control | 0.1 | 0.25 | 0.5 | 1.0 | 1.5 |
| PGE (pg/ml) | 62 ± 24 | 53 ± 18 | 45 ± 12 | 44 ± 11 | 32 ± 8 | 30 ± 9 |
| 6-keto-$PGF_{1\alpha}$ (pg/ml) | 88 ± 30 | 125 ± 35 | 170 ± 27 | 219 ± 24 | 417 ± 54* | 780 ± 147* |
| PAF (ng/ml) | 1.0 ± 0.3 | 4.4 ± 0.5 | 10.1 ± 2.6 | 13.1 ± 2.6* | 20.8 ± 5.3* | 22.4 ± 5.0* |
| LDH (mU/L) | 25 ± 3 | 39 ± 7 | 86 ± 16* | 89 ± 13* | 78 ± 17* | 89 ± 9* |

Each value represents the mean ± S.E.M. of five values obtained from the mean of duplicate levels from five gall-bladders. An asterisk indicates that the value is significantly different from control. $1 \times 10^6$ mucosal cells were incubated in buffer solution for 1 h alone and with varying concentrations of LPC.

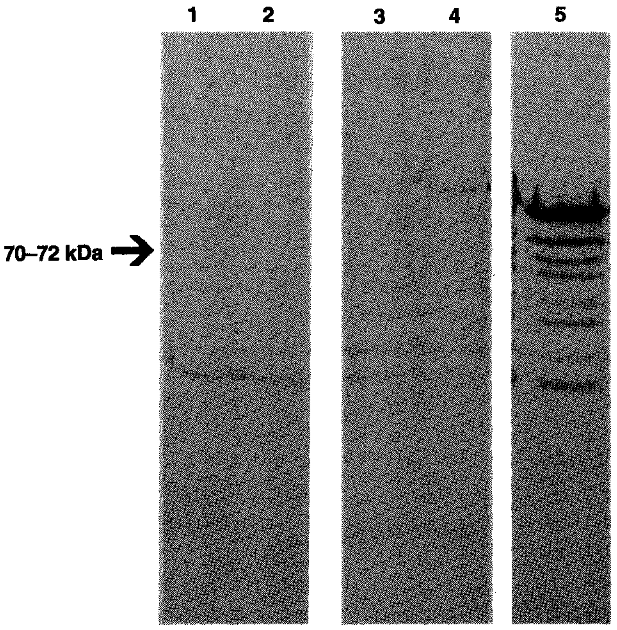

FIG. 1. Evaluation of microsomal COX-1 expression by unstimulated and LPC-treated human gall-bladder mucosal cells. Lanes 1 and 2 represent 15 ng of microsomal protein from control buffer treated cells with no LPC and Lanes 3 and 4 represent 15 ng of protein from cells treated with LPC. In three separate gall-bladder specimens, COX-1 was not detectable or only barely detectable. Lane 5 is COX-1 protein as positive control.

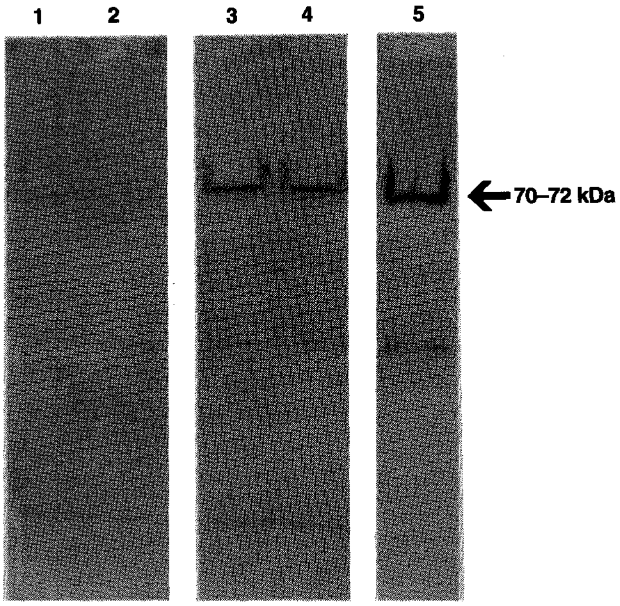

FIG. 2. Effect of LPC on microsomal COX-2 protein expression. Human gall-bladder mucosal cells were treated with 0.5 mM LPC for 1 h. The microsomal protein was prepared and analysed by Western blotting. Each lane represents protein from mucosal cells from a single gall-bladder. Lanes 1 and 2 represent 15 μg of microsomal protein from control, buffer treated cells with no LPC and Lanes 3 and 4 represent 15 μg of protein from cells treated with LPC immunoblotted with rabbit anti-COX-2 antibody. The molecular weight marker identifies the 70–72 kDa COX protein. Lane 5 represents 100 ng of protein as a positive control. In three gall-bladder specimens COX-2 protein expression was induced by LPC.

Western blot analysis was unable to detect COX-1 protein in normal or inflamed skin, while COX-2 expression was increased by pro-inflammatory agents.[22] Similarly, in human endothelial cells COX-1 expression was not detected employing Northern blot analysis unless a sensitive reverse transcription, polymerase chain reaction assay was employed.[23,24] Determination of the relative contributions of COX-1 and COX-2 to prostanoid formation in stimulated and unstimulated gall-bladder mucosal cells will require continued evaluation with LPC and other pro-inflammatory agents.

In previous studies in cats, experimental cholecystitis was produced by lipopolysaccharide administration.[15] Human gall-bladder mucosal cells exposed to lipopolysaccharide produce large amounts of PAF and prostanoids.[11] Interestingly, the pro-inflammatory stimuli lipopolysaccharide and LPC produce relatively specific changes in arachidonic acid metabolism. Lipopolysaccharide did not change 6-keto-$PGF_{1\alpha}$ production by human gall-bladder mucosal cells, while the stimulus markedly increased PGE production. As indicated in the present study, LPC produced primarily increased prostacyclin formation.

In intact animals, it is possible to produce a severe tissue destructive, inflammatory disorder in the gall-bladder that mimics the inflammatory disorder in humans.[7,9,15,25] In isolated human gall-bladder mucosal cells it is presently unclear what causes the cellular damage evident in cholecystitis. As PAF produced a remarkable degree of tissue destruction *in vivo*,[9] it was felt that this substance, if produced by gall-bladder cells by increased phospholipase $A_2$

activity, may contribute to or produce the cell lysis. As is evident in this study and as found previously with lipopolysaccharide,[11] human gall-bladder mucosal cells exposed to large amounts of intra and extracellular PAF remain physically intact. As the question is relevant not only to cholecystitis, but to other gastrointestinal inflammatory disorders as well, further studies will be needed in order to clarify the nature of the tissue destructive agents. While phospholipase $A_2$ and COX activity may be relevant in developing the inflammatory cascade of events, it seems unlikely that their products cause the cellular damage. Other potential factors that may contribute to the tissue destruction include ischaemia, ischaemia/reperfusion,[26] nitric oxide,[27] or factors produced by leukocytes and macrophages.[28]

### References

1. Carey MC, Cahalane MJ. Whither biliary sludge? *Gastroenterology* 1988; **95**: 508–523.
2. Sjodahl R, Wetterfors J. Lysolecithin and lecithin in gallbladder wall and bile: their possible role in the pathogenesis of acute cholecystitis. *Scand J Gastroenterol* 1974; **9**: 519–525.
3. Gottfries A, Nilsson S, Samuelsson B, Scheraten T. Phospholipids in human hepatic bile, gallbladder bile and plasma in cases with acute cholecystitis. *Scand J Lab Clin Invest* 1968; **21**: 168–176.
4. Weltzien HU. Cytolytic and membrane-perturbing properties of lysophosphatidylcholine. *Biochim Biophys Acta* 1979; **559**: 259–287.
5. Gottfried A. Lysolecithin a factor in the pathogenesis of acute cholecystitis. *Acta Chir Scand* 1969; **135**: 213–217.
6. La Morte WW, LaMont JT, Habe W, Booker ML, Scott TE, Turner B. Gallbladder prostaglandins and lysophospholipids as mediators of mucin secretion during cholelithiasis. *Am J Physiol* 1986; **251**: G701–G709.
7. Kaminski DL, Dean D, Deshpande YG. Prostanoids and leukotrienes in experimental feline cholecystitis. *Hepatology* 1990; **11**: 1003–1009.
8. Snyder F. Platelet-activating factor and related acetylated lipids as potent biologically active cellular mediators. *Am J Physiol* 1990; **259**: C697–C708.
9. Kaminski DL, Andrus CH, German D, Deshpande YG. The role of prostanoids in the production of acute acalculous cholecystitis by platelet-activating factor. *Ann Surg* 1990; **212**: 455–461.
10. Mayer RJ, Marshall LA. New insights on mammalian phospholipase $A_2$(s); comparison of arachidonoyl-selective and non-selective enzymes. *FASEB J* 1993; **7**: 339–348.
11. Kaminski DL, Amir G, Deshpande YG, Beck D, Li AP. Studies on the etiology of acute acalculous cholecystitis: the effect of lipopolysaccharide on human gallbladder mucosal cells. *Prostaglandins* 1994; **47**: 319–330.
12. Yoshitomi S, Miyazuki K, Makayama F. Demonstration and maintenance of mucous secretion in cultured human gallbladder epithelial cells. *In Vitro Cell Dev Biol* 1987; **28**: 559–565.
13. Neiderhiser D, Thornell E, Bjorck S, Svanik J. The effect of lysophosphatidylcholine on gallbladder function in the cat. *J Lab Clin Med* 1983; **101**: 699–707.
14. Bradford MM. A rapid and sensitive method for the quantitation of microgram quantities of protein utilizing the principle of protein-dye binding. *Anal Biochem* 1976; **72**: 248–254.
15. Kaminski DL, Feinstein WK, Deshpande YG. The production of experimental cholecystitis by endotoxin. *Prostaglandins* 1994; **47**: 233–245.
16. Estrada C, Gomez C, Martin C, Moncada S, Gonzales C. Nitric oxide mediates tumor necrosis factor-α cytotoxicity in endothelial cells. *Biochem Biophys Res Comm* 1992; **186**: 475–482.
17. Laemmli UK. Cleavage of structural proteins during the assembly of the head of the bacteriophage. *Nature* 1970; **227**: 680–685.
18. Matsudaira PT, Burgess DR. SDS microslab linear gradient polyacrylamide gel electrophoresis. *Anal Biochem* 1978; **87**: 386–396.
19. Wong WYL, De Witt DL, Smith WL, Richards JS. Rapid induction of prostaglandin endoperoxide synthase in rat preovulatory follicles by luteinizing hormone and cAMP is blocked by inhibitors of transcription and translation. *Molec Endocrinol* 1989; **3**: 1714–1723.
20. O'Sullivan MG, Huggins EM, Meade EA, De Witt DL, McCall CE. Lipopolysaccharide induces prostaglandin H synthase-2 in alveolar macrophages. *Biochem Biophys Res Comm* 1992; **187**: 1123–1127.
21. Ohno K, Fugimoto M, Hirata M. Protective effect of prostaglandin $H_2$ against menadione-induced cell injury in cultured porcine aorta endothelial cells. *Chem Biol Interactions* 1991; **78**: 67–75.
22. Vane JR, Mitchell JA, Appleton I, Tomlinson A, Bishop-Bailey D, Croxtall J, Willoughby DA. Inducible isoforms of cyclooxygenase and nitric-oxide synthase in inflammation. *Proc Natl Acad Sci* 1994; **91**: 2046–2050.
23. Maier JAM, Hla T, Maciag T. Cyclooxygenase is an immediate early gene induced by interleukin-1 in human endothelial cells. *J Biol Chem* 1990; **265**: 10805–10808.
24. Hla T. Regulation of cyclooxygenase gene expression in vascular endothelial cells. In: Bailey JM, ed. *Prostaglandins, Leukotrienes, Lipoxins and PAF.* New York: Plenum Press, 1991; 53.
25. German D, Barcia J, Brems J, Merendez G, Kaminski DL. Effect of bradykinin on feline gallbladder water transport and prostanoid formation. *Dig Dis Sci* 1989; **34**: 1770–1776.
26. Taoka H. Experimental study on the pathogenesis of acute acalculous cholecystitis, with special reference to the roles of microcirculatory disturbances, free radicals, and membrane-bound phospholipase $A_2$. *Gastroenterol J* 1991; **26**: 633–644.
27. Ialenti A, Ianaro A, Moncada S, Di Rosa M. Modulation of acute inflammation by endogenous nitric oxide. *Eur J Pharmacol* 1992; **211**: 197–182.
28. Williams TJ, Hellewell PG. Adhesion molecules involved in the microvascular inflammatory response. *Am Rev Res Dis* 1992; **146**: 545–550.

ACKNOWLEDGEMENT. This work was supported by USPHS Grant DDK 27695.

**Received 16 August 1994; accepted in revised form 7 November 1994**
